# The Roles of CCR9/CCL25 in Inflammation and Inflammation-Associated Diseases

**DOI:** 10.3389/fcell.2021.686548

**Published:** 2021-08-19

**Authors:** Xue Wu, Meng Sun, Zhi Yang, Chenxi Lu, Qiang Wang, Haiying Wang, Chao Deng, Yonglin Liu, Yang Yang

**Affiliations:** ^1^Department of Paediatrics, Shenmu Hospital, School of Life Sciences and Medicine, Northwest University, Shenmu, China; ^2^Key Laboratory of Resource Biology and Biotechnology in Western China, Ministry of Education, Faculty of Life Sciences, Northwest University, Xi’an, China; ^3^Department of Cardiology, The First Hospital of Shanxi Medical University, Taiyuan, China; ^4^Department of Cardiovascular Surgery, The First Affiliated Hospital of Xi’an Jiaotong University, Xi’an, China

**Keywords:** chemokine, CCR9, CCL25, autoimmune, inflammatory disease

## Abstract

Chemokine is a structure-related protein with a relatively small molecular weight, which can target cells to chemotaxis and promote inflammatory response. Inflammation plays an important role in aging. C-C chemokine receptor 9 (CCR9) and its ligand C-C chemokine ligand 25 (CCL25) are involved in the regulating the occurrence and development of various diseases, which has become a research hotspot. Early research analysis of CCR9-deficient mouse models also confirmed various physiological functions of this chemokine in inflammatory responses. Moreover, CCR9/CCL25 has been shown to play an important role in a variety of inflammation-related diseases, such as cardiovascular disease (CVD), rheumatoid arthritis, hepatitis, inflammatory bowel disease, asthma, etc. Therefore, the purpose of this review gives an overview of the recent advances in understanding the roles of CCR9/CCL25 in inflammation and inflammation-associated diseases, which will contribute to the design of future experimental studies on the potential of CCR9/CCL25 and advance the research of CCR9/CCL25 as pharmacological inflammatory targets.

## Introduction

**FIGURE 1:**
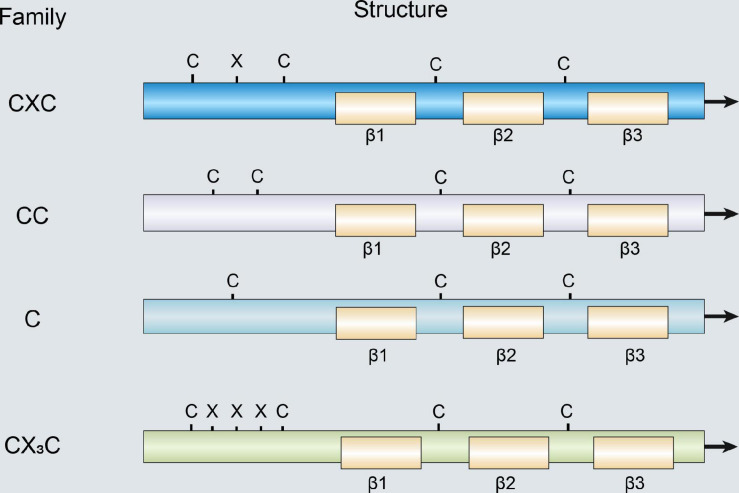
Structure of members of the chemokine family.

Chemokines are a large family of small cytokines and generally have low molecular weight ranging from 7 to 15 kDa (Palomino and Marti, 2015). Chemokines can interact with different chemokine receptors that control the residence and migration of immune cells. When the body is stimulated by antigen, chemokines can be secreted by various cells such as endothelial and dendritic cells (DCs) (Vassilatis et al., 2003). Currently, more than 50 chemokines have been discovered, which can be divided into four categories according to the position of two cysteines at the N-terminus: C subfamily, CC subfamily, CXC subfamily, and CX3C subfamily (Figure 1; Bleul et al., 1996). Moreover, according to the expression of chemokines and their functions in the immune system, chemokines can be also divided into homeostatic and inflammatory chemokines (Table 1; Moser et al., 2004). Chemokines expression is regulated via a variety of factors such as interleukin (IL)-10 and glucocorticoids, which can inhibit the secretion of chemokines, while IL-1 and tumor necrosis factor (TNF)-α can increase chemokines expression (Cocchi et al., 1995; Oberlin et al., 1996; Zlotnik and Yoshie, 2012). The chemokine receptor is a G-protein coupled receptor of the seven-transmembrane receptor family, which contains seven transmembrane segments and is widely expressed on white blood cells surface derived from bone marrow such as neutrophils and macrophages (Schulz et al., 2016). In addition, chemokine receptors have also been shown to be expressed in epithelial cells, vascular endothelial cells, and other tissue cells. The combination of chemokines and their corresponding receptors can involve in normal body development, and also participate in various pathological and physiological processes such as leukocyte activation, angiogenesis, aging, breast cancer resistance, and autoimmune diseases (Zhong et al., 2015).

**TABLE 1:** Classification, structural features, function, and location of the chemokine family members.

Family	Structural features	Function	Location	Chemokines
CXC	The CXC family has a single amino acid residue in between the first two cysteines.	CXC chemokines have the highest ability to attract neutrophils and monocytes.	4q12-13 4q21.21 10q11.1 4q21 5p31 17p13	CXCL1-8 CXCL9-11 CXCL12 CXCL13 CXCL14 CXCL1
CC	The CC chemokine family has the first two cysteine residues adjacent to each other.	CC chemokines are mainly responsible for the recruitment of lymphocytes.	17q11.2 7q11.23 16q13 17q11.2 9q13 2q33-3 9q13 16q13 11q11.2 7q11.23 19p13.2 7q11.23 9q13 5q	CCL1-15 CCL16 CCL17 CCL18 CCL19 CCL20 CCL21 CCL22 CCL23 CCL24 CCL25 CCL26 CCL27 CCL28
C	The C family there is only one cysteine residue and only one disulfide chain.	C chemokine has chemotactic activity for lymphocytes, CD4^+^ and CD8^+^ T cells, and NK cells.	1q23	XCL1-2
CX3C	The CX3C chemokine family three amino acid residues separate the first two cysteines.	CX3C chemokines can induce chemotaxis of monocytes and cytotoxic T cells.	16q13	CX3CL1

*The spacing of two conserved cysteine residues at the N-terminus determines the nomenclature and classification of the chemokine family members; in CXC, CC, C, and CX3C chemokines. Moreover, according to the classification of chemokines, these four types also have different functions and localizations.*

C-C chemokine ligand 25 (CCL25) is also considered a thymus expressed chemokine, and the gene is located on chromosome 19p13.2 (Figure 1; Qiuping et al., 2004). Tu et al. (2016) confirmed that CCL25 is mainly expressed in the thymus and intestinal epithelium, and also produced by vascular endothelial cells and other parenchymal cells, which can migrate immature T cells into the thymus to mature and release (Table 1). C-C chemokine receptor 9 (CCR9) was discovered in 1996 as a G protein-coupled receptors expressed on the cell membrane of dendritic cell, neutrophils, lymphocytes, monocyte macrophages, and vascular endothelial cells (Yu and Stamenkovic, 2000). However, in the 3 years after the discovery of CCR9, its ligand has not been found, so it was once called "orphan receptor." It was not until 1999 that CCL25 was found to be one-to-one combined with CCR9 (Zaballos et al., 1999). CCR9 belongs to the β-chemokine receptor family, and its gene is located on chromosome 3 p21.31 (Tu et al., 2016). CCR9 is mainly distributed in immature T lymphocytes and intestinal cells surface, and after binding to its specific ligands, plays a key role in T lymphocyte development and tissue-specific homing (Wermers et al., 2011; Wu et al., 2014). Further study found that CCL25 promoted proliferation and chemotaxis of inflammatory cells that expressed its specific receptor CCR9 (Igaki et al., 2018).

**FIGURE 2:**
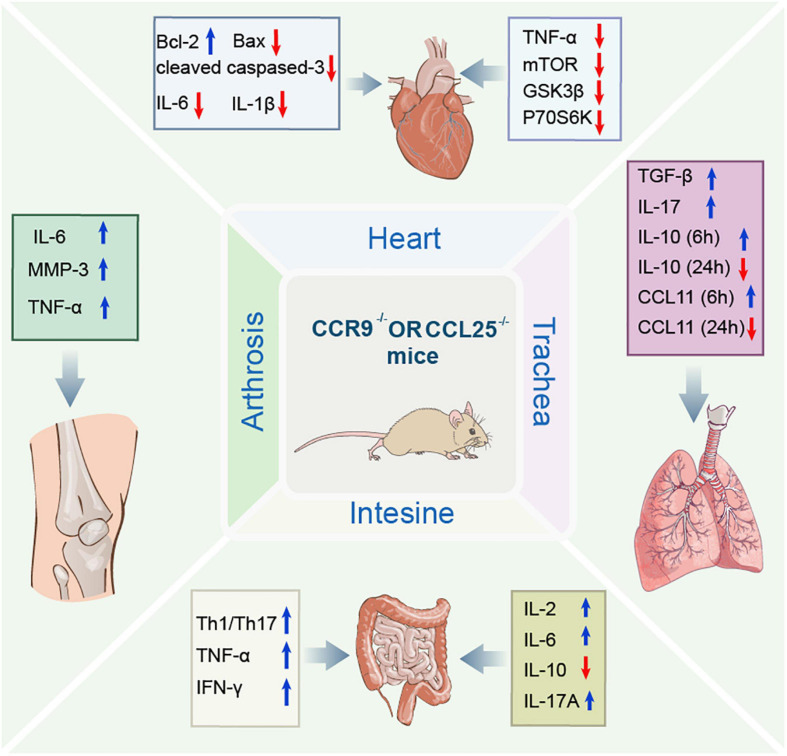
Schematic diagram illustrating the regulation of molecular mechanisms of CCR9/CCL25 in inflammatory diseases. CCR9/CCL25 exerts multiple effects in a variety of inflammatory diseases by regulating molecular mechanisms, such as molecules associated with apoptosis and inflammation. CCR9, C-C chemokine receptor 9; CCL25, C-C chemokine ligand 25; Bcl-2, B-cell lyumphoma-2; Bax, Bcl-2 Associated X-protein; IL-6, interleukin-6; IL-1β, interleukin-1β; TNF-α, tumor necrosis factor-α; mTOR, mechanistic target of rapamycin; GSK3β, glycogen synthase kinase 3-beta; p70S6K, p70 S6 kinase; IL-10, interleukin-10; MMP3, matrix metalloproteinase-3; TNF-β, tumor necrosis factor-β; IL-17, interleukin-17; CCL11, C-C chemokine ligand 11; Th1/Th17, helper T1/helper T17; IFN-γ, interferon-gamma; IL-2, interleukin-2; IL-17A, interleukin-17A.

CCR9 and CCL25 are members of the CC subfamily of chemokines that are involved in a variety of inflammatory diseases and promote inflammatory responses. Over the years, the role of CCR9/CCL25 in inflammation and related diseases has become increasingly clearness, including cardiovascular disease (CVD), hepatitis, arthritis (Yokoyama et al., 2014), inflammatory bowel disease (Kalindjian et al., 2016), and asthma. This study provides abundant evidence that CCR9/CCL25 can be a potential target for a variety of inflammatory diseases. Therefore, the focus of this review is to evaluate the latest findings on the role of CCR9/CCL25 in inflammatory disease. The structure and physiological function of CCR9/CCL25 were briefly introduced. The various roles of CCR9/CCL25 in the development of inflammatory disease were described (Figure 2), and some different insights were put forward concerning the CCR9/CCL25 pathway.

## CCR9 and CCL25 Expression in Tissue

Vicari et al. (1997) isolated CCL25 cDNA from the thymus of recombinase activation gene-1 (RAG-1) deficient mice and were designated thymus-expressed chemokine (TECK). Moreover, they also found that CCL25 appeared weak sequence homology to other members of the CC chemokine family and located on mouse chromosome 8 (Vicari et al., 1997). Besides the thymus, mRNA encoding CCL25 was detected at substantial levels in the small intestine and at low levels in the liver (Vicari et al., 1997; Zabel et al., 1999; Kunkel et al., 2000). However, studies found that CCL25 is highly expressed in the small intestinal epithelium and thymus, which can regulate trafficking of gut-specific memory/effector T cells via upregulation of the integrin homing receptor α47 and CCR9 (Stenstad et al., 2006; Wurbel et al., 2000, 2007). Meanwhile, the source of CCL25 in the thymus was determined to be thymic dendritic cells; in contrast, bone marrow-derived dendritic cells do not express CCL25. The murine CCL25 recombinant protein showed chemotactic activity for activated macrophages, dendritic cells, and thymocytes. These research results fully demonstrated that CCL25 represents a novel thymic dendritic cell-specific CC chemokine that is possibly involved in T cell development. Within the intestines of normal mice, CCL25 expression was highest in the proximal small intestine, lowest in the distal small bowel, and no or almost no expression in the colon (Svensson et al., 2002; Rivera-Nieves et al., 2006; Stenstad et al., 2007; Wermers et al., 2011; Wurbel et al., 2011).

In circulating white blood cells, CCR9 expression is limited to activated B cells and a certain proportion of CD4 and CD8 T cells (Zabel et al., 1999; Kunkel et al., 2000; Papadakis et al., 2000, 2001, 2003). CCR9 has been identified in plasmacytoid dendritic cells in mice, but there is a lack of relevant data in humans (Wendland et al., 2007; Hadeiba et al., 2012). Similarly, despite the fact that a few papers describe the expression of CCR9 on mouse macrophages, there is a lack of human data (Nakamoto et al., 2012). In the peripheral tissues, CCR9^+^ cells are mostly concentrated in colon, thymus, and small intestine (Zabel et al., 1999; Kunkel et al., 2000; Papadakis et al., 2000). Intraepithelial lymphocytes are mainly CD8^+^, most of which express CCR9 surface (Zabel et al., 1999; Kunkel et al., 2000).

## The Role of CCR9/CCL25 in Inflammatory Diseases

Inflammation underlies many physiological and pathological processes. In particular, chronic inflammation is attributed to the pathophysiological basis of various modern diseases. Of note, inflammatory diseases are characterized by wide coverage, complex pathogenesis, and large difference in prognosis (Guo et al., 2015). Therefore, we mainly focus on the effect of CCR9/CCL25 in inflammatory diseases as well as elaborate on their mechanism of action in related diseases.

### Cardiovascular Disease

Cardiovascular disease, also known as circulatory disease, is a series of diseases involving the circulatory system, including the heart and blood vessels, which have always been at the forefront of human major causes of death (Jokinen, 2015). Heart failure, cardiac hypertrophy, and atherosclerosis are belong to CVD and are also a chronic vascular inflammatory disease, which have high morbidity, high mortality, and high disability rate, and thus, seriously threaten human health (Van Camp, 2014).

In CCR9 knockout CCR9^+/+^ mice, surgical ligation of the left anterior descending coronary artery caused myocardial infarction (MI), and found that the expression of CCR9 in the heart of mice was significantly up-regulated after MI. Down-regulation of CCR9 expression can improve survival rate and left ventricular dysfunction, reduce infarct size, and improve cardiac function after MI. In addition, abolish of CCR9 in the mouse MI heart significantly increased Bcl-2 expression, while the expression of Bax and cleaved caspase 3 was remarkably reduced, thereby attenuating the apoptosis of cardiomyocytes. Inflammation is considered the most vital pathological response to damage and repair, and abolish of CCR9 can decrease pro-inflammatory cytokines mRNA levels (IL-6, IL-1β, and TNF-α) and suppress the inflammatory response after MI. In addition, CCR9 is involved in structural remodeling mainly by interfering with NF-κB and MAPK signaling pathways (Figure 2). These results confirmed that CCR9/CCL25 may exert a positive role in MI (Huang et al., 2016).

Heart failure is the leading cause of death in many countries, particularly in an aging population (Kumarswamy and Thum, 2013). Currently, heart failure is widely recognized as a clinical syndrome caused by cardiac hypertrophy and remodeling that involves changes of structural and functional in the left ventricle (Zhang et al., 2014). Study found that CCR9 protein levels have significantly increased in failing human hearts and mice or cardiomyocyte hypertrophy model. The loss of CCR9 in mice can reduce the hypertrophy caused by pressure overload. The results show that heart weight/body weight, lung weight, and heart weight/tibia length ratios of CCR9-deficient mice are significantly lower than those of the control group. And aortic banding treated CCR9-deficient mice were characterized by decreased Left ventricular (LV) end diastolic and systolic diameters and increased fractional shortening, demonstrating smaller LV dimensions and elevated systolic function. Moreover, the hypertrophic response of the CCR9-deficient mice was remarkably attenuated compared with controls, and these animals manifested decreased heart size and cardiomyocyte cross sectional area as well as alleviation of interstitial fibrosis. The mRNA levels of hypertrophic and fibrotic markers were also reduced significantly in the CCR9 deficient mice. Notably, overexpression of CCR9 in mice reversed the result of the appeal (Xu et al., 2016). In terms of mechanism, they further found that the expression levels of MAPK lack difference between the two groups, while the phosphorylation levels of AKT/protein kinase B and downstream effectors (mTOR, GSK3b, and p70S6K) were remarkably reduced in CCR9 knockout mice and increased in CCR9 transgenic mice after aortic surgery (Figure 2). These results suggested that CCR9 could promote hypertrophy mainly due to the protein kinase B mammalian target of the rapamycin GSK-3β signaling cascade, rather than through the MAPK signaling pathway, which indicates that CCR9 can be used as a new regulator of myocardial hypertrophy and may provide a novel therapeutic target for suppressing myocardial hypertrophy in the future (Xu et al., 2016).

In conclusion, the knockout of CCR9/CCL25 serves as a novel modulator of pathological progression for inhibiting the development of MI and heart failure (Table 2). However, the role of CCR9/CCL25 in other CVD has not been reported, so further research is needed.

**TABLE 2:** The role of CCR9/CCL25 in inflammatory-associated diseases.

Inflammation disease	Expression of CCR9 or CCL25	Model	Effect of CCR9/CCL25	Outcome	References
Myocardial infarction	Increased CCR9	Model of myocardial infarction induced by ligation of left anterior descending coronary artery of CCR9^–/–^ and CCR9^+/+^ mice	Down-regulation of CCR9 expression can improve survival rate, left ventricular dysfunction, cardiac function, reduce infarct size, meanwhile, significantly attenuate the apoptosis of cardiomyocytes, and inhibit the inflammatory response after myocardial infarction.	CCR9/CCL25 axis may play an important role in inflammatory cell infiltration and cardiac remodeling after myocardial infarction.	Huang et al., 2016
Hepatitis	–	Concanavalin A-induced murine acute liver injury and human acute hepatitis	Macrophages expressing CCR9^+^CD11b^+^ aggravated liver damage through production of inflammatory cytokines and the promotion of Th1 cell development.	Inflammatory macrophages originated from bone marrow and became locally differentiated and proliferated by interaction with hepatic stellate cells via knocking out CCR9 gene during acute liver injury.	Amiya et al., 2016; Nakamoto, 2016
Rheumatoid arthritis	Increased CCR9 and CCL25	In the synovium of RA and non-RA patients	Stimulation with CCL25 increased IL-6 and MMP-3 production from RA fibroblast-like synoviocytes, and also increased IL-6 and TNF-α production from peripheral blood monocytes. Collagen-induced arthritis was suppressed in CCR9-deficient mice.	The interaction between CCL25 and CCR9 may promote cell infiltration and production of inflammatory mediator in RA synovial tissues.	Yokoyama et al., 2014
Inflammatory bowel disease	Increased CCR9 and CCL25	DSS-induced acute colitis	CCR9^–/–^ mice are more susceptible to DSS colitis than WT littermate controls as shown by higher mortality, increased IBD score, and delayed recovery. During recovery, the CCR9^–/–^ colonic mucosa is characterized by the accumulation of activated macrophages and elevated levels of Th1/Th17 inflammatory cytokines.	CCR9/CCL25 interaction regulates the inflammatory immune response of the intestinal mucosa by balancing different dendritic cell subsets.	Wurbel et al., 2011
Inflammatory bowel disease	Increased CCR9 and CCL25	DSS-induced acute colitis	The mice lacking the interaction of CCR9/CCL25 showed enhanced adhesion promoting ability of coli after the transfer of naive T cells, and regulated the pro-inflammatory and anti-inflammatory subgroups of cDC.	CCR9/CCL25 interactions have protection against large intestinal inflammation in chronic colitis.	Wurbel et al., 2014
Asthma	Increased CCL25	OVA-induced model of allergic inflammation within CCR9 deficient mice	In challenged CCR9-deficient mice, cell recruitment was impaired at peribronchial and perivenular levels. OVA administration in CCR9-deficient mice leads to a less inflammatory cell recruitment, which modifies the expression of IL-10, CCL11, and CCL25 after OVA challenge.	CCR9 and CCL25 expressions are induced in the early stages of airway inflammation and they have an important role in modulating eosinophils and lymphocytes recruitment at the first stages of inflammatory process.	Lopez-Pacheco et al., 2016
Asthma	Increased CCR9	NKT cells	In patients with allergic asthma, the CCR9/CCL25 binding method selectively induces chemotaxis of NKT cells. And NKT cells may be in direct contact with CD3^+^ T cells, polarizing them to a Th2 bias.	CCR9/CCL25 signal can interact with CD226 signaling to activate asthmatic NKT cells, leading to airway hyperresponsiveness and inflammation, aggravating asthma.	Sen et al., 2005

*Compelling evidence has indicated that alterations of CCR9 or CCL25 expression are also involved in inflammatory-associated diseases, including myocardial infarction, hepatitis, rheumatoid arthritis, inflammatory bowel disease, and asthma.*

*CCR9, C-C chemokine receptor 9; CCL25, C-C chemokine ligand 25; RA, rheumatoid arthritis; IBD, inflammatory bowel disease; DSS, dextran sulfate sodium; DCs, dendritic cells; IL-6, interleukin-6; IL-10, interleukin-10; MMP3, matrix metalloproteinase-3; TNF-α, tumor necrosis factor-α; NKT, natural killer T; DCs, dendritic cells; Th, helper T.*

### Hepatitis

Hepatitis usually refers to various pathogenic factors, including parasites, viruses, drugs, bacteria, and autoimmune factors, which cause damage to liver cells and functions, thereby causing a series of uncomfortable symptoms of the body and abnormal liver function indicators (Crispe, 2003; Thomson and Knolle, 2010; Linder and Malani, 2017).

As well known, various immune cells in the liver involve in the pathogenesis of liver diseases. Inflammatory macrophages play a key role in liver injury and then liver fibrosis and canceration. Nakamoto et al. (2012) previous research suggests that CCR9^+^ inflammatory macrophage triggers acute liver injury by interacting with helper T 1 (Th1) cells in the inflamed liver. Subsequently, they found that macrophages expressing CCR9^+^CD11b^+^ aggravated liver damage through production of inflammatory cytokines and the promotion of Th1 cell development during the concanavalin A-induced murine acute liver injury and human acute hepatitis (Nakamoto, 2016). Further analysis using liver-shielded radiation and bone marrow transplantation mouse models revealed that these CCR9^+^CD11b^+^ macrophages were originated from bone marrow-derived monocytes, but not liver resident macrophages. In addition, in contact with hepatic stellate cells, these CD11b^+^ inflammatory macrophages participated in the pathogenesis of experimental liver fibrosis by knocking out CCR9 gene. The results with further verification in human samples clarified the pathogenic role of CCR9/CCL25 axis as therapeutic target of a variety of liver diseases (Nakamoto, 2016). Similarly, Amiya et al. (2016) also demonstrated that in acute liver injury, inflammatory macrophages originated from bone marrow and became locally differentiated and proliferation through interaction with hepatic stellate cells by knocking out CCR9 gene (Figure 2).

Primary sclerosing cholangitis (PSC), as a chronic inflammatory liver disease, is characterized by progressive bile duct destruction and is also a parenteral complication of inflammatory bowel disease (IBD). In addition, PSC is a non-suppurative autoimmune granulomatous inflammation of the small bile ducts in the liver that can lead to chronic progressive cholestasis and eventually cirrhosis and liver failure (Chapman, 1991). In the healthy liver, the expression of CCL25 is very low or even undetected (Vicari et al., 1997; Zabel et al., 1999; Eksteen et al., 2004). However, in PSC, the expression of CCL25 was increased in the liver, mainly through hepatic sinusoidal endothelial cells and portal DCs expression (Eksteen et al., 2004). The hepatic inflammation in PSC was related to the increase of CCR9 positive T cells. Other study proposed that long-lived memory gut homing cells that expressed CCR9 and α_4_β_7_ and were early activated during episodes of IBD could exacerbate PSC through interactions with hepatic endothelial that ectopically expressed CCL25 (Eksteen et al., 2004). Notably, they also demonstrated for the first time that T cells activated in the intestine can be recruited to extra-intestinal site of disease in humans and provide basic research to explain the pathogenesis of extra-intestinal complications of IBD (Eksteen et al., 2004; Table 2).

### Rheumatoid Arthritis

Rheumatoid arthritis (RA) is a chronic inflammatory disease that can cause a large number of macrophages, T cells, and B cells to accumulate in the synovium (Firestein, 1991; Kinne et al., 2000), and the accumulation of these cells can further participate in the development of inflammation, joint destruction, and pain. However, biological agents (TNF blockers and IL-6 receptor antagonists) are effective in RA patients (Feldmann, 2002; Nishimoto et al., 2004; Smolen et al., 2010).

A large number of mononuclear/macrophages accumulate in the rheumatoid synovium, which play a promotive role in inflammation and joint destruction. Identifying the molecules involved in its accumulation and differentiation is essential for the development of therapeutic strategies (Mulherin et al., 1996; Haringman et al., 2005; Nishimoto and Takagi, 2010). Cluster of differentiation (CD) 14 and CD68 co-localize with CCR9 or CCL25 to identify macrophages. Other studies have confirmed that the augment CD68^+^ cells in the substratum of RA are one of the characteristic pathological changes of synovial membrane in early RA (Singh et al., 2004). Other study demonstrated that CCR9 was expressed by PB monocytes/macrophages in RA and healthy donors, and increased in RA. Moreover, in the synovium of RA and non-RA patients, CCR9 colocates with CD14^+^ and CD68^+^ macrophages and was more abundant in RA synovium. Therefore, the percentage of CCR9^+^ monocytes in the synovium of RA (81%) is increased compared to that in blood (40%), and the percentage of CCR9^+^ monocytes in the synovium of non-RA (66%) is greater than that in blood (16%). Meanwhile, CCL25 could be detected in both RA and non-RA synovia, which can be co-localized within CD14^+^ and CD68^+^ cells (Schmutz et al., 2010). Furthermore, CCL25 induced stronger monocyte differentiation in RA. CCL25 induced significant chemotaxis of peripheral blood monocytes, however, which was inconsistent between different individuals. These results indicate that monocyte-induced CCR9/CCL25 expression is significantly increased in RA. CCL25 may be involved in the differentiation of monocytes to macrophages particularly in RA (Haringman et al., 2005). Moreover, CCR9 and CCL25 are expressed at higher levels in RA synovial tissues compared to osteoarthritis synovial tissues. Most CD68^+^ macrophages in RA synovial tissue express CCR9 and CCL25 revealed by immunohistochemical. CCR9 was expressed in macrophage, fibroblast-like synoviocytes, and DCs of synovial tissues. CCL25 stimulated the production of IL-6 and MMP-3 in RA fibroblast-like synoviocytes, and also increased the production of IL-6 and TNF-α in peripheral blood monocytes (Figure 2). Collagen-induced arthritis was inhibited in CCR9^–/–^ mice. CCR9 antagonist (CCX8037) also suppressed collagen-induced arthritis and decreased the migration of CD11b^+^ splenic cells to synovial tissues. These results suggested that the interaction between CCL25 and CCR9 may promote cell infiltration and production of inflammatory mediator in RA synovial tissues. Blocking CCL25 or CCR9 may represent a strange new safety therapy for RA, and provide theoretical basis for further research a novel safe therapy for RA (Yokoyama et al., 2014; Table 2).

### Inflammatory Bowel Disease

Inflammatory bowel disease is a group of chronic, non-specific inflammatory bowel diseases whose etiology has not yet been clarified, mainly including ulcerative colitis (UC) and Crohn’s disease (CD). UC is a continuous inflammation of the colonic mucosa and submucosa that first affects the rectum and gradually spreads to the entire colon. CD can affect the entire digestive tract and is a discontinuous full-layer inflammation, most commonly involving terminal ileum, colon, and perianal (Loftus, 2004; Molodecky et al., 2012).

Wurbel et al. (2011) study the acute inflammation and recovery in WT and CCR9^–/–^ mice in a model of dextran sulfate sodium (DSS)-induced colitis. The results show that CCL25 and CCR9 are both expressed in the large intestine and are upregulated during DSS colitis. CCR9^–/–^ mice are more susceptible to DSS colitis than WT littermate controls as shown by higher mortality, increased IBD score, and delayed recovery. During recovery, the CCR9^–/–^ colonic mucosa is characterized by the accumulation of activated macrophages and added Th1/Th17 inflammatory cytokines levels. Activated plasmacytoid dendritic cells (pDC) accumulate in the mesenteric lymph nodes of CCR9^–/–^ animals, changing the local proportion of DC subsets. The T cells separate from these mesenteric lymph nodes were stimulated again, which were significantly higher levels of TNF-α, IFN-γ, IL-2, IL-6, and IL-17A while down-regulating IL-10 production (Figure 2). These results suggest that the CCR9/CCL25 interaction regulates the inflammatory immune response of the intestinal mucosa by balancing different dendritic cell subsets (Wurbel et al., 2011).

As mentioned before, CCR9^–/–^ and CCL25^–/–^ mice are more susceptible to acute DSS colitis than WT controls. As human ulcerative colitis is associated with signs of chronic colonic inflammation, they investigated whether the increased susceptibility to acute inflammation associated with defective CCL25/CCR9 interactions would also translate into increased susceptibility to chronic inflammation, and found that chronic DSS exposure results in exacerbated colitis in mice deficient for either CCR9 or CCL25 when compared with WT control mice. Although CCR9^–/–^ T cells traffic to the colon and induce severe colitis similar to WT T cells, naive WT T cells induce more severe disease in recipient animals devoid of CCL25 expression. Moreover, compared with WT control mice, there was no significant difference in the total number of pDC and conventional dendritic cells (cDC), and the pro-inflammatory and anti-inflammatory subgroups of cDC were regulated by the CCR9/CCL25 interaction. In the end, the CCR9/CCL25-dependent innate immune cell lineage specificity and lineage-dependent function will be remarkably assisted via targeting the loss of CCR9/CCL25 in innate immune cell and/or epithelial compartment. These results suggested that CCR9/CCL25 interactions have protection against large intestinal inflammation in chronic colitis (Wurbel et al., 2014). Moreover, Papadakis et al. (2001) demonstrated that the proportion of peripheral blood CCR9^+^ CD4^+^ cells rose remarkably in active small bowel CD compared with normal groups, but not in patients with purely colonic Crohn’s. It is worth noting that GSK/Chemocentryx has advanced its CCR9 antagonist CCX282-B (also known as vercirnon) to a pivotal phase III clinical trial. It was unfortunate that due to poor efficacy of IBD, the clinical project was terminated at the end of 2013 (Zhang et al., 2015).

The pathophysiological characteristics of necrotizing enterocolitis (NEC) include excessive inflammation and necrosis, which can affect any part of the gastrointestinal tract (especially the small intestine) (Neu and Walker, 2011; Nino et al., 2016). The imbalance caused by the decrease in tolerogenic Foxp3+ regulatory T (Treg) cells and the increase in the production of Th17 cells in the lamina propria pro-inflammatory il-17 leads to the NEC-induced excessive inflammatory response (Weitkamp et al., 2013; Liu et al., 2014; Egan et al., 2016). Moreover, another study has shown that in the peripheral blood of NEC patients and mice, the proportion of CCR9^+^ CD4^+^ T cells was significantly elevated. Increased CCR9^+^ CD4^+^ T cells were mainly CCR9^+^ IL-17-producing Treg cells, which are characteristic of common Treg cells, but their inhibitory activities were seriously damaged and passively correlated with the serious of intestinal tissue injury. Treg cells that produce CCR9^+^ IL-17 may be an important biomarker, improving NEC by regulating the balance of lymphocytes (Ma et al., 2019). In summary, these results demonstrated that CCR9^+^ IL-17-producing Treg cells could be an important biomarker, but further research is needed for clinical use (Table 2).

### Asthma

Asthma, as a lung disease, is characterized by reversible airway obstruction, airway inflammation, and increased airway responsiveness to a variety of stimuli (Hahn, 2009; Mims, 2015). Chemokine receptors have been confirmed to be involved in leukocyte recruitment, and are closely related to asthma pathology (DeKruyff et al., 2014; Griffith et al., 2014). An ovalbumin (OVA)-induced allergic inflammation model was established in CCR9^–/–^ mice, and the expression of CCR9 and CCL25 in eosinophils and T lymphocytes was found 6 h post-OVA challenge. Meanwhile, compared with wild-type mice, the peribronchial infiltration in CCR9-deficient mice was significantly reduced (nearly 50%), while the total number of eosinophils recruited in bronchoalveolar fluid (BALF) was decreased (30%). Moreover, in CCR9^–/–^ mice, OVA administration can reduce the recruitment of inflammatory cell, which modifies IL-10, CCL11, and CCL25 expression at 24 h after OVA (Lopez-Pacheco et al., 2016). Interestingly, expression of TGF-β and IL-17 was elevated in OVA-stimulated CCR9-deficient mice compared to WT mice, whereas IL-10, as an anti-inflammatory chemokine, was significantly reduced (more than 60%). In addition, there was an increase in the expression of CCL25 in the WT mice as early as 6 h after ova-challenge. Meanwhile, CCL25 expression was decreased at all time test in CCR9^–/–^ mice (Figure 2). The above results confirm that CCR9 deficiency has a positive regulatory effect on eosinophils and lymphocytes in the early stage of inflammation induction, suggesting that they may be potential targets for regulating asthma inflammation in asthma (Lopez-Pacheco et al., 2016).

Natural killer T (NKT) cells are the main operators in the development of asthma. Multiple NKT cells migrate and aggregate in the airway, producing a Th2 bias effect that directly or indirectly promotes asthma. Studies have found that the recruitment of NKT cells depends on the high expression of CCR9 and the connection of CCR9/CCL25 (DeKruyff et al., 2014; Griffith et al., 2014). In patients with allergic asthma, the CCR9/CCL25 binding method selectively induces chemotaxis of NKT cells, whereas in healthy volunteers, normal NKT cells are not able to induce. Further studies have confirmed that the pathways for NKT cells regulate the development of asthma. In the process of migrating from blood vessels to airway bronchial mucosa, NKT cells may directly contact CD3^+^ T cells, making them polarized toward Th2. This regulatory function depends on DC participation and CCR9/CD226 coordinated activation. The adhesion molecule CD226 is overexpressed in asthmatic NKT cells. The CCR9/CCL25 linkage can directly phosphorylate CD226, and the lack of CD226 can block the Th2 bias effect induced by NKT cells. These results indicate that CCR9/CCL25 signaling pathway could interact with CD226 signals to activate asthmatic NKT cells, leading to airway hyperresponsiveness and inflammation, aggravating asthma (Sen et al., 2005). In addition, Castan et al. (2018) demonstrated that CCR9 knock-out mice eliminated the aggravation of lung symptoms in consecutive food and respiratory allergies, which were featured by a rose in lung resistance and a higher Th17/Treg ratio in solely asthmatic-like mice. Moreover, to better understand the mechanism underlying the food allergy-induced aggravation of asthma and the role of CCR9, they performed adoptive transfer of CD4^+^ T cells from food-allergic mice into naïve mice, which were subsequently sensitized to house dust mites. They found that asthmatic mice received food-sensitized CD4^+^ T cells and had more severe inflammation than asthmatic mice that received non-sensitized CD4^+^ T cells. Interestingly, when mice received food allergen-sensitized lymphocytes from CCR9^–/–^ mice, the exacerbation of asthma caused by food allergen CD4^+^ lymphocytes was eliminated. These results show that CCR9 is a driving factor for the worsening of lung inflammation in food allergic mice (Castan et al., 2018), and confirm its application potential in the development of treatment strategies for allergic diseases (Table 2).

### Autoimmune Diseases

Toll-like receptor 4 (TLR4) is an important part of innate immunity and has been linked to central nervous system (CNS) inflammatory diseases (Ureña-Peralta et al., 2020). Zhang et al. (2019) have shown that TLR4^–/–^ mice were inadequate to induce experimental autoimmune encephalomyelitis (EAE), which is featured by reducing low clinic score, weight loss, demyelinating, and spinal cord inflammatory cell infiltration. The deletion of TLR4 in the lesion area of EAE mice can decrease inflammatory cytokines and CCL25 secretion. After transformation, CCR9 expression is reduced in TLR4^–/–^ Th17 cells, and the chemotaxis and migration ability are weakened. In summary, TLR4 may play an important role in the infiltration of Th17 during the pathogenesis of EAE by CCL25/CCR9 signaling pathway (Zhang et al., 2019). Kadowaki et al. (2019) also demonstrate that in the mice model of experimental autoimmune encephalomyelitis, CCR9^+^ memory T cells preferentially infiltrate the inflammatory CNS and express lymphocyte activation 3 gene (LAG3) in the late stage. Antibiotic treatment can alleviate the symptoms of experimental autoimmune encephalomyelitis, which is supported by a significant rise in peripheral blood CCR9^+^ memory T cells. Generally speaking, they postulate that the alterations in CCR9^+^ memory T cells observed, caused by either the gut microbiota changes or ageing, may lead to the development of secondary progressive multiple sclerosis (Kadowaki et al., 2019). Moreover, the CCL25/CCR9 was also powerful to authorize effector/memory T cells to access anatomic sites (Evans-Marin et al., 2015). Binding of CCR9 to CCL25 inhibits CD4 T cells differentiating to Tregs. In autoimmunity, CCR9^+^ T helper cells induce diabetes by secreting IL-21 and promote the expansion and survival of CD8t cells (McGuire et al., 2011).

Omenn syndrome is resulted from a mild rag mutation, which is featured by severe immunodeficiency and similar manifestations of autoimmunity (Villa et al., 1998; Khiong et al., 2007). Rigoni et al. (2016) confirmed that long-term use of broad-spectrum antibiotics in Rag2 (R229Q) mice can ameliorate intestinal and systemic autoimmunity by reducing mucosal and circulating gut-tropic CCR9(+) Th1 and Th17 T cells frequency. In addition, during the allergic reaction, CCL25 drives the mobilization of IL-17^+^ γδ T cells to inflamed tissues through α4β7 integrin, and regulates the level of IL-17 (Costa et al., 2012). The above studies have proved that CCR9/CCL25 plays an important role in autoimmune diseases.

### Cancer

In microenvironment, tumor formation and immune system are mutually restricted, and chemokines also play an important role between tumor formation and immune system (Fan et al., 2019; Rossi et al., 2019; Lei et al., 2020). T cells are one of the main functional cells of the anti-cancer immune response. CD4^+^ T cells have an important regulatory effect, and CD8^+^ T cell stem cell-like subgroups have the potential to self-proliferate and differentiate toward effector cells, which can produce durable anti-tumor immune response and specifically kill tumor cells (Borst et al., 2018; Farhood et al., 2019; He et al., 2019; Brightman et al., 2020).

Studies have found that T cell function depends on the expression of CCR9. CD4^+^ CCR9^+^ T helper cells express high amounts of interleukin 21 and induce T cell costimulatory factor, transcription factor B cell CLL/lymphoma 6 (Bcl-6), and Maf, which are local features of autoimmune diseases that affect the accessory organs of the digestive system (McGuire et al., 2011). The CCR9 signal transmitted during the startup of naïve T cells promotes the differentiation of memory CD4^+^ T cells that produce α 4 β + 7 IFN-γ, increasing the immune microenvironment in gastrointestinal tissues, thereby affecting effector immunity in infections and cancer (Fu et al., 2019). Notably, Chen et al. (2020) used tumor acidity-responsive nanoparticle delivery system (NP-siCD47/CCL25) to discharge CCL25 protein and CD47 siRNA in tumors to enhance CD47 targeted immunotherapy.

In the study, NP-siCD47/CCL25 remarkably increased the invasiveness of CCR9^+^CD8^+^ T cells, inhibited the expression of CD47 in tumor cells, and inhibited tumor cells metastasis and growth by T-cell-dependent immunity (Chen et al., 2020). In addition, Khandelwal et al. (2015) pointed out that CCR9 regulates the STAT signal in T cells and inhibits the secretion and cytotoxicity of T lymphocytes type 1 cytokines. This study shows that inhibiting CCR9 expression may promote tumor-specific T cell immunotherapy (Khandelwal et al., 2015). In conclusion, targeting CCR9/CCL25 is expected to be a new approach for treating tumors, by suppressing or treating tumor patients with an autoimmune system that produces a lasting antitumor response.

## Conclusion and Potential Future Directions

Inflammatory diseases have become a serious threat to the health of the global population. Inflammatory diseases involve lesions of multiple tissues and organs in the body, which are characterized by tissue damage caused by excessive or persistent inflammation, such as CVD, hepatitis, asthma, RA, and inflammatory bowel diseases. However, the common features of these diseases are abnormal regulation of inflammatory response and imbalance of immune pathways. Chemokines are a family of small molecule cytokines whose receptors are seven-transmembrane glycoproteins, mainly expressed on the surface of inflammatory cells. Previous studies have shown that as members of the chemokine CC subfamily, CCR9 and CCL25 have been related to various inflammatory diseases as well as could promote inflammatory responses. Recently, the roles of CCR9/CCL25 in inflammation and related diseases have become clearer. CCR9/CCL25 is participated in many inflammatory diseases, including CVD, hepatitis, RA, IBD, and asthma (Table 2). Although the functional studies regarding CCR9/CCL25 in inflammatory diseases have gradually deepened, the understanding of CCR9/CCL25 has become more comprehensive. However, studies on the mechanisms of CCR9/CCL25 underlying inflammatory diseases are mostly restricted to the theoretical basic, and their clinical application is few.

Although several lines of evidence have clarified the specific mechanism of CCR9/CCL25 signaling in inflammation-associated diseases, the development of successful treatment of CCR9/CCL25 is still in further study stage. CCX282-B is bioavailable in the circulation following oral administration, and which has high specificity for CCR9 with negligible binding to any other chemokine receptor, have been evaluated in murine models of IBD as well as in clinical trials. Meanwhile, related studies have confirmed that the use of an oral antagonist for CCR9 (CCX282-B) was also evaluated in phase II and phase III clinical trials with mixed results, but the results were not as expected. Therefore, to make further progress in delineating the potential clinical role of CCR9 in human disease, detailed mechanistic studies and well-designed proof-of-concept human trials are now required. Further investigation is likely to focus on (1) studying the effects of blocking CCR9 on the activation state of circulating and tissue-infiltrating lymphocytes, (2) investigating disease remission in CD remains a strong indication for CCR9 antagonist treatment, and any future clinical trials should include patients with UC and with PSC, (3) investigating whether the clinical application of CCR9 antagonism has any suboptimum efficacy, and (4) continuing to study CCR9/CCL25 upstream and downstream molecules, which are possible future potential therapeutic targets for inflammatory diseases. In conclusion, this review presents a comprehensive picture of the role of CCR9/CCL25 in inflammatory diseases and proposes a new underlying therapeutic mechanisms and target for the future treatment of inflammatory diseases.

## Author Contributions

XW and MS: conceptualization, literature search, and original draft writing and editing. ZY, CL, QW, HW, and CD: original draft writing and editing. YL and YY: conceptualization, original draft writing and editing, supervision, and funding acquisition. All authors contributed to the article and approved the submitted version.

## Conflict of Interest

The authors declare that the research was conducted in the absence of any commercial or financial relationships that could be construed as a potential conflict of interest.

## Publisher’s Note

All claims expressed in this article are solely those of the authors and do not necessarily represent those of their affiliated organizations, or those of the publisher, the editors and the reviewers. Any product that may be evaluated in this article, or claim that may be made by its manufacturer, is not guaranteed or endorsed by the publisher.

## References

[B1] Amiya T. Nakamoto N. Chu P. S. Teratani T. Nakajima H. Fukuchi Y. (2016). Bone marrow-derived macrophages distinct from tissue-resident macrophages play a pivotal role in Concanavalin A-induced murine liver injury via CCR9 axis. *Sci Rep.* 6 35146. 10.1038/srep35146 PMC5057133 27725760

[B2] Bleul C. C. Farzan M. Choe H. Parolin C. Clark-Lewis I. Sodroski J. (1996). The lymphocyte chemoattractant SDF-1 is a ligand for LESTR/fusin and blocks HIV-1 entry. *Nature.* 382 829–833. 10.1038/382829a0 8752280

[B3] Borst J. Ahrends T. Ba̧bała N. Melief C. J. M. Kastenmüller W. (2018). CD4(+) T cell help in cancer immunology and immunotherapy. *Nat Rev. Immunol.* 18 635–647. 10.1038/s41577-018-0044-0 30057419

[B4] Brightman S. E. Naradikian M. S. Miller A. M. Schoenberger S. P. (2020). Harnessing neoantigen specific CD4 T cells for cancer immunotherapy. *J Leukoc. Biol.* 107 625–633. 10.1002/jlb.5ri0220-603rr 32170883 PMC7793607

[B5] Castan L. Cheminant M. A. Colas L. Brouard S. Magnan A. Bouchaud G. (2018). Food allergen-sensitized CCR9(+) lymphocytes enhance airways allergic inflammation in mice. *Allergy* 73 1505–1514. 10.1111/all.13386 29315632

[B6] Chapman R. W. (1991). Aetiology and natural history of primary sclerosing cholangitis–a decade of progress? *Gut.* 32 1433–1435. 10.1136/gut.32.12.1433 1773944 PMC1379237

[B7] Chen H. Cong X. Wu C. Wu X. Wang J. Mao K. (2020). Intratumoral delivery of CCL25 enhances immunotherapy against triple-negative breast cancer by recruiting CCR9(+) T cells. *Science Advances* 6 4690. 10.1126/sciadv.aax4690 PMC6989134 32064335

[B8] Cocchi F. DeVico A. L. Garzino-Demo A. Arya S. K. Gallo R. C. Lusso P. (1995). Identification of RANTES, MIP-1 alpha, and MIP-1 beta as the major HIV-suppressive factors produced by CD8+ T cells. *Science.* 270 1811–1815. 10.1126/science.270.5243.1811 8525373

[B9] Costa M. F. Bornstein V. U. Candéa A. L. Henriques-Pons A. Henriques M. G. Penido C. (2012). CCL25 induces α*4*β*7* integrin-dependent migration of IL-17^+^ γδ T lymphocytes during an allergic reaction. *Eur J. Immunol.* 42 1250–1260. 10.1002/eji.201142021 22539297

[B10] Crispe I. N. (2003). Hepatic T cells and liver tolerance. *Nat Rev. Immunol.* 3 51–62. 10.1038/nri981 12511875

[B11] DeKruyff R. H. Yu S. Kim H. Y. Umetsu D. T. (2014). Innate immunity in the lung regulates the development of asthma. *Immunol. Rev.* 260 235–248. 10.1111/imr.12187 24942693

[B12] Egan C. E. Sodhi C. P. Good M. Lin J. Jia H. Yamaguchi Y. (2016). Toll-like receptor 4-mediated lymphocyte influx induces neonatal necrotizing enterocolitis. *J Clin. Invest.* 126 495–508. 10.1172/jci83356 26690704 PMC4731173

[B13] Eksteen B. Grant A. J. Miles A. Curbishley S. M. Lalor P. F. Hubscher S. G. (2004). Hepatic endothelial CCL25 mediates the recruitment of CCR9+ gut-homing lymphocytes to the liver in primary sclerosing cholangitis. *J Exp. Med.* 200 1511–1517. 10.1084/jem.20041035 15557349 PMC2211943

[B14] Evans-Marin H. L. Cao A. T. Yao S. Chen F. He C. Liu H. (2015). Unexpected Regulatory Role of CCR9 in Regulatory T Cell Development. *PLoS One.* 10:e0134100. 10.1371/journal.pone.0134100 26230654 PMC4521878

[B15] Fan L. Li Y. Chen J. Y. Zheng Y. F. Xu X. M. (2019). Immune checkpoint modulators in cancer immunotherapy: Recent advances and combination rationales. *Cancer Lett.* 456 23–28. 10.1016/j.canlet.2019.03.050 30959079

[B16] Farhood B. Najafi M. Mortezaee K. (2019). CD8(+) cytotoxic T lymphocytes in cancer immunotherapy: A review. *J Cell Physiol.* 234 8509–8521. 10.1002/jcp.27782 30520029

[B17] Feldmann M. (2002). Development of anti-TNF therapy for rheumatoid arthritis. *Nat Rev. Immunol.* 2 364–371. 10.1038/nri802 12033742

[B18] Firestein G. S. (1991). The immunopathogenesis of rheumatoid arthritis. *Curr Opin. Rheumatol.* 3 398–406. 10.1097/00002281-199106000-00012 1883694

[B19] Fu H. Jangani M. Parmar A. Wang G. Coe D. Spear S. (2019). A Subset of CCL25-Induced Gut-Homing T Cells Affects Intestinal Immunity to Infection and Cancer. *Front Immunol.* 10:271. 10.3389/fimmu.2019.00271 PMC6400137 30863398

[B20] Griffith J. W. Sokol C. L. Luster A. D. (2014). Chemokines and chemokine receptors: positioning cells for host defense and immunity. *Annu Rev. Immunol.* 32 659–702. 10.1146/annurev-immunol-032713-120145 24655300

[B21] Guo H. Callaway J. B. Ting J. P. (2015). Inflammasomes: mechanism of action, role in disease, and therapeutics. *Nat. Med.* 21 677–687. 10.1038/nm.3893 26121197 PMC4519035

[B22] Hadeiba H. Lahl K. Edalati A. Oderup C. Habtezion A. Pachynski R. (2012). Plasmacytoid dendritic cells transport peripheral antigens to the thymus to promote central tolerance. *Immunity.* 36 438–450. 10.1016/j.immuni.2012.01.017 22444632 PMC3315699

[B23] Hahn D. L. (2009). Importance of evidence grading for guideline implementation: the example of asthma. *Ann. Fam Med.* 7 364–369. 10.1370/afm.995 19597175 PMC2713157

[B24] Haringman J. J. Gerlag D. M. Zwinderman A. H. Smeets T. J. Kraan M. C. Baeten D. (2005). Synovial tissue macrophages: a sensitive biomarker for response to treatment in patients with rheumatoid arthritis. *Ann Rheum. Dis.* 64 834–838. 10.1136/ard.2004.029751 15576415 PMC1755544

[B25] He Q. F. Xu Y. Li J. Huang Z. M. Li X. H. Wang X. (2019). CD8+ T-cell exhaustion in cancer: mechanisms and new area for cancer immunotherapy. *Brief. Funct Genomics.* 18 99–106. 10.1093/bfgp/ely006 29554204

[B26] Huang Y. Wang D. Wang X. Zhang Y. Liu T. Chen Y. (2016). Abrogation of CC chemokine receptor 9 ameliorates ventricular remodeling in mice after myocardial infarction. *Sci Rep.* 6 32660. 10.1038/srep32660 PMC5009347 27585634

[B27] Igaki K. Komoike Y. Nakamura Y. Watanabe T. Yamasaki M. Fleming P. (2018). MLN3126, an antagonist of the chemokine receptor CCR9, ameliorates inflammation in a T cell mediated mouse colitis model. *Int. Immunopharmacol.* 60 160–169. 10.1016/j.intimp.2018.04.049 29730559

[B28] Jokinen E. (2015). Obesity and cardiovascular disease. *Minerva. Pediatr.* 67 25–32. 25387321

[B29] Kadowaki A. Saga R. Lin Y. Sato W. Yamamura T. (2019). Gut microbiota-dependent CCR9+CD4+ T cells are altered in secondary progressive multiple sclerosis. *Brain.* 142 916–931. 10.1093/brain/awz012 30770703 PMC6439331

[B30] Kalindjian S. B. Kadnur S. V. Hewson C. A. Venkateshappa C. Juluri S. Kristam R. (2016). A New Series of Orally Bioavailable Chemokine Receptor 9 (CCR9) Antagonists; Possible Agents for the Treatment of Inflammatory Bowel Disease. *J Med. Chem.* 59 3098–3111. 10.1021/acs.jmedchem.5b01840 26987013

[B31] Khandelwal N. Breinig M. Speck T. Michels T. Kreutzer C. Sorrentino A. (2015). A high-throughput RNAi screen for detection of immune-checkpoint molecules that mediate tumor resistance to cytotoxic T lymphocytes. *EMBO. Mol. Med.* 7 450–463. 10.15252/emmm.201404414 25691366 PMC4403046

[B32] Khiong K. Murakami M. Kitabayashi C. Ueda N. Sawa S. Sakamoto A. (2007). Homeostatically proliferating CD4 T cells are involved in the pathogenesis of an Omenn syndrome murine model. *J Clin. Invest.* 117 1270–1281. 10.1172/jci30513 17476359 PMC1857265

[B33] Kinne R. W. Brauer R. Stuhlmuller B. Palombo-Kinne E. Burmester G. R. (2000). Macrophages in rheumatoid arthritis. *Arthritis. Res.* 2 189–202. 11094428 10.1186/ar86 PMC130001

[B34] Kunkel E. J. Campbell J. J. Haraldsen G. Pan J. Boisvert J. Roberts A. I. (2000). Lymphocyte CC chemokine receptor 9 and epithelial thymus-expressed chemokine (TECK) expression distinguish the small intestinal immune compartment: Epithelial expression of tissue-specific chemokines as an organizing principle in regional immunity. *J Exp. Med.* 192 761–768. 10.1084/jem.192.5.761 10974041 PMC2193265

[B35] Lei X. Lei Y. Li J. K. Du W. X. Li R. G. Yang J. (2020). Immune cells within the tumor microenvironment: Biological functions and roles in cancer immunotherapy. *Cancer. Lett.* 470 126–133. 10.1016/j.canlet.2019.11.009 31730903

[B36] Liu Y. Tran D. Q. Fatheree N. Y. Marc Rhoads J. (2014). Lactobacillus reuteri DSM 17938 differentially modulates effector memory T cells and Foxp3+ regulatory T cells in a mouse model of necrotizing enterocolitis. *Am J. Physiol. Gastrointest. Liver Physiol.* 307 G177–G186. 24852566 10.1152/ajpgi.00038.2014 PMC4101683

[B37] Loftus E. V. Jr. (2004). Clinical epidemiology of inflammatory bowel disease: Incidence, prevalence, and environmental influences. *Gastroenterology.* 126 1504–1517. 10.1053/j.gastro.2004.01.063 15168363

[B38] Lopez-Pacheco C. Soldevila G. Du Pont G. Hernandez-Pando R. Garcia-Zepeda E. A. (2016). CCR9 Is a Key Regulator of Early Phases of Allergic Airway Inflammation. *Mediators Inflamm* 2016 3635809. 10.1155/2016/3635809 PMC5067335 27795621

[B39] Ma F. Li S. Gao X. Zhou J. Zhu X. Wang D. (2019). Interleukin-6-mediated CCR9(+) interleukin-17-producing regulatory T cells polarization increases the severity of necrotizing enterocolitis. *EBioMedicine.* 44 71–85. 10.1016/j.ebiom.2019.05.042 31129099 PMC6604880

[B40] Malani P. N. (2017). Hepatitis A. *JAMA.* 318 2393. 10.1001/jama.2017.17244 29094153

[B41] Marti L. C. (2015). Chemokines and immunity. *Einstein (São Paulo).* 13 469–473. 26466066 10.1590/S1679-45082015RB3438 PMC4943798

[B42] McGuire H. M. Vogelzang A. Ma C. S. Hughes W. E. Silveira P. A. Tangye S. G. (2011). A subset of interleukin-21+ chemokine receptor CCR9+ T helper cells target accessory organs of the digestive system in autoimmunity. *Immunity.* 34 602–615. 10.1016/j.immuni.2011.01.021 21511186

[B43] Mims J. W. (2015). Asthma: definitions and pathophysiology. *Int Forum. Allergy Rhinol.* 5 Suppl 1 S2–S6. 26335832 10.1002/alr.21609

[B44] Molodecky N. A. Soon I. S. Rabi D. M. Ghali W. A. Ferris M. Chernoff G. (2012). Increasing incidence and prevalence of the inflammatory bowel diseases with time, based on systematic review. *Gastroenterology* 142 46–54.e42; quiz e30. 22001864 10.1053/j.gastro.2011.10.001

[B45] Moser B. Wolf M. Walz A. Loetscher P. (2004). Chemokines: multiple levels of leukocyte migration control. *Trends. Immunol.* 25 75–84. 10.1016/j.it.2003.12.005 15102366

[B46] Mulherin D. Fitzgerald O. Bresnihan B. (1996). Synovial tissue macrophage populations and articular damage in rheumatoid arthritis. *Arthritis. Rheum.* 39 115–124. 10.1002/art.1780390116 8546720

[B47] Nakamoto N. (2016). Role of inflammatory macrophages and CCR9/CCL25 chemokine axis in the pathogenesis of liver injury as a therapeutic target. *Nihon Rinsho. Meneki. Gakkai Kaishi.* 39 460–467. 10.2177/jsci.39.460 27795503

[B48] Nakamoto N. Ebinuma H. Kanai T. Chu P. S. Ono Y. Mikami Y. (2012). CCR9+ macrophages are required for acute liver inflammation in mouse models of hepatitis. *Gastroenterology.* 142 366–376. 10.1053/j.gastro.2011.10.039 22079594

[B49] Nino D. F. Sodhi C. P. Hackam D. J. (2016). Necrotizing enterocolitis: new insights into pathogenesis and mechanisms. *Nat Rev Gastroenterol Hepatol.* 13 590–600. 10.1038/nrgastro.2016.119 27534694 PMC5124124

[B50] Nishimoto N. Takagi N. (2010). Safety and efficacy profiles of tocilizumab monotherapy in Japanese patients with rheumatoid arthritis: meta-analysis of six initial trials and five long-term extensions. *Mod Rheumatol.* 20 222–232. 10.3109/s10165-010-0279-5 20221663

[B51] Nishimoto N. Yoshizaki K. Miyasaka N. Yamamoto K. Kawai S. Takeuchi T. (2004). Treatment of rheumatoid arthritis with humanized anti-interleukin-6 receptor antibody: a multicenter, double-blind, placebo-controlled trial. *Arthritis Rheum.* 50 1761–1769. 10.1002/art.20303 15188351

[B52] Oberlin E. Amara A. Bachelerie F. Bessia C. Virelizier J. L. Arenzana-Seisdedos F. (1996). The CXC chemokine SDF-1 is the ligand for LESTR/fusin and prevents infection by T-cell-line-adapted HIV-1. *Nature.* 382 833–835. 10.1038/382833a0 8752281

[B53] Papadakis K. A. Landers C. Prehn J. Kouroumalis E. A. Moreno S. T. Gutierrez-Ramos J. C. (2003). CC chemokine receptor 9 expression defines a subset of peripheral blood lymphocytes with mucosal T cell phenotype and Th1 or T-regulatory 1 cytokine profile. *J. Immunol.* 171 159–165. 10.4049/jimmunol.171.1.159 12816994

[B54] Papadakis K. A. Prehn J. Moreno S. T. Cheng L. Kouroumalis E. A. Deem R. (2001). CCR9-positive lymphocytes and thymus-expressed chemokine distinguish small bowel from colonic Crohn’s disease. *Gastroenterology.* 121 246–254. 10.1053/gast.2001.27154 11487533

[B55] Papadakis K. A. Prehn J. Nelson V. Cheng L. Binder S. W. Ponath P. D. (2000). The role of thymus-expressed chemokine and its receptor CCR9 on lymphocytes in the regional specialization of the mucosal immune system. *J. Immunol.* 165 5069–5076. 10.4049/jimmunol.165.9.5069 11046037

[B56] Qiuping Z. Jei X. Youxin J. Wei J. Chun L. Jin W. (2004). CC chemokine ligand 25 enhances resistance to apoptosis in CD4+ T cells from patients with T-cell lineage acute and chronic lymphocytic leukemia by means of livin activation. *Cancer Res.* 64 7579–7587. 10.1158/0008-5472.can-04-0641 15492285

[B57] Rigoni R. Fontana E. Guglielmetti S. Fosso B. D’Erchia A. M. Maina V. (2016). Intestinal microbiota sustains inflammation and autoimmunity induced by hypomorphic RAG defects. *J Exp. Med.* 213 355–375. 10.1084/jem.20151116 26926994 PMC4813669

[B58] Rivera-Nieves J. Ho J. Bamias G. Ivashkina N. Ley K. Oppermann M. (2006). Antibody blockade of CCL25/CCR9 ameliorates early but not late chronic murine ileitis. *Gastroenterology.* 131 1518–1529. 10.1053/j.gastro.2006.08.031 17101325

[B59] Rossi J. F. Céballos P. Lu Z. Y. (2019). Immune precision medicine for cancer: a novel insight based on the efficiency of immune effector cells. *Cancer Commun. (Lond).* 39 34. 10.1186/s40880-019-0379-3 31200766 PMC6567551

[B60] Schmutz C. Cartwright A. Williams H. Haworth O. Williams J. H. Filer A. (2010). Monocytes/macrophages express chemokine receptor CCR9 in rheumatoid arthritis and CCL25 stimulates their differentiation. *Arthritis Res. Ther.* 12 R161. 10.1186/ar3120 PMC2945064 20738854

[B61] Schulz O. Hammerschmidt S. I. Moschovakis Forster R. (2016). Chemokines and Chemokine Receptors in Lymphoid Tissue Dynamics. *Annu Rev. Immunol.* 34 203–242. 10.1146/annurev-immunol-041015-055649 26907216

[B62] Sen Y. Yongyi B. Yuling H. Luokun X. Li H. Jie X. (2005). V alpha 24-invariant NKT cells from patients with allergic asthma express CCR9 at high frequency and induce Th2 bias of CD3+ T cells upon CD226 engagement. *J. Immunol.* 175 4914–4926. 10.4049/jimmunol.175.8.4914 16210593

[B63] Singh J. A. Pando J. A. Tomaszewski J. Schumacher H. R. (2004). Quantitative analysis of immunohistologic features of very early rheumatoid synovitis in disease modifying antirheumatic drug- and corticosteroid-naive patients. *J. Rheumatol.* 31 1281–1285. 15229944

[B64] Smolen J. S. Landewe R. Breedveld F. C. Dougados M. Emery P. Gaujoux-Viala C. (2010). EULAR recommendations for the management of rheumatoid arthritis with synthetic and biological disease-modifying antirheumatic drugs. *Ann Rheum, Dis.* 69 964–975. 20444750 10.1136/ard.2009.126532 PMC2935329

[B65] Yu Q. Stamenkovic I. (2000). Cell surface-localized matrix metalloproteinase-9 proteolytically activates TGF-beta and promotes tumor invasion and angiogenesis. *Genes. Dev.* 14 163–176. 10652271 PMC316345

[B66] Stenstad H. Ericsson A. Johansson-Lindbom B. Svensson M. Marsal J. Mack M. (2006). Gut-associated lymphoid tissue-primed CD4+ T cells display CCR9-dependent and -independent homing to the small intestine. *Blood.* 107 3447–3454. 10.1182/blood-2005-07-2860 16391017

[B67] Stenstad H. Svensson M. Cucak H. Kotarsky K. Agace W. W. (2007). Differential homing mechanisms regulate regionalized effector CD8alphabeta+ T cell accumulation within the small intestine. *Proc Natl Acad Sci U S A.* 104 10122–10127. 10.1073/pnas.0700269104 17551016 PMC1891238

[B68] Svensson M. Marsal J. Ericsson A. Carramolino L. Broden T. Marquez G. (2002). CCL25 mediates the localization of recently activated CD8alphabeta(+) lymphocytes to the small-intestinal mucosa. *J Clin. Invest.* 110 1113–1121. 10.1172/jci0215988 12393847 PMC150799

[B69] Thomson A. W. Knolle P. A. (2010). Antigen-presenting cell function in the tolerogenic liver environment. *Nat. Rev. Immunol.* 10 753–766. 10.1038/nri2858 20972472

[B70] Thum T. (2013). Non-coding RNAs in cardiac remodeling and heart failure. *Circ. Res.* 113 676–689. 10.1161/circresaha.113.300226 23989712

[B71] Tu Z. Xiao R. Xiong J. Tembo K. M. Deng X. Xiong M. (2016). CCR9 in cancer: oncogenic role and therapeutic targeting. *J Hematol. Oncol.* 9 10. 10.1186/s13045-016-0236-7 PMC4754913 26879872

[B72] Ureña-Peralta J. R. Pérez-Moraga R. García-García F. (2020). Lack of TLR4 modifies the miRNAs profile and attenuates inflammatory signaling pathways. *PLoS One* 15:e0237066. 10.1371/journal.pone.0237066 32780740 PMC7418977

[B73] Van Camp G. (2014). Cardiovascular disease prevention. *Acta Clin. Belg.* 69 407–411. 25176558 10.1179/2295333714Y.0000000069

[B74] Vassilatis D. K. Hohmann J. G. Zeng H. Li F. Ranchalis J. E. Mortrud M. T. (2003). The G protein-coupled receptor repertoires of human and mouse. *Proc Natl Acad Sci U S A.* 100 4903–4908. 12679517 10.1073/pnas.0230374100 PMC153653

[B75] Vicari A. P. Figueroa D. J. Hedrick J. A. Foster J. S. Singh K. P. Menon S. (1997). TECK: a novel CC chemokine specifically expressed by thymic dendritic cells and potentially involved in T cell development. *Immunity.* 7 291–301. 10.1016/s1074-7613(00)80531-2 9285413

[B76] Villa A. Santagata S. Bozzi F. Giliani S. Frattini A. Imberti L. (1998). Partial V(D)J recombination activity leads to Omenn syndrome. *Cell.* 93 885–896. 10.1016/s0092-8674(00)81448-8 9630231

[B77] Walker W. A. (2011). Necrotizing enterocolitis. *N Engl. J. Med.* 364 255–264. 21247316 10.1056/NEJMra1005408 PMC3628622

[B78] Weitkamp J. H. Koyama T. Rock M. T. Correa H. Goettel J. A. Matta P. (2013). Necrotising enterocolitis is characterised by disrupted immune regulation and diminished mucosal regulatory (FOXP3)/effector (CD4, CD8) T cell ratios. *Gut.* 62 73–82. 10.1136/gutjnl-2011-301551 22267598 PMC3606820

[B79] Wendland M. Czeloth N. Mach N. Malissen B. Kremmer E. Pabst O. (2007). CCR9 is a homing receptor for plasmacytoid dendritic cells to the small intestine. *Proc Natl Acad Sci U S A.* 104 6347–6352. 10.1073/pnas.0609180104 17404233 PMC1851094

[B80] Wermers J. D. McNamee E. N. Wurbel M. A. Jedlicka P. Rivera-Nieves J. (2011). The chemokine receptor CCR9 is required for the T-cell-mediated regulation of chronic ileitis in mice. *Gastroenterology* 140 1526–1535.e1523. 21300065 10.1053/j.gastro.2011.01.044 PMC3086928

[B81] Wu W. Doan N. Said J. Pullarkat S. T. (2014). Strong expression of chemokine receptor CCR9 in diffuse large B-cell lymphoma and follicular lymphoma strongly correlates with gastrointestinal involvement. *Hum Pathol.* 45 1451–1458. 10.1016/j.humpath.2014.02.021 24828696

[B82] Wurbel M. A. Le Bras S. Ibourk M. Pardo M. McIntire M. G. Coco D. (2014). CCL25/CCR9 interactions are not essential for colitis development but are required for innate immune cell protection from chronic experimental murine colitis. *Inflamm Bowel. Dis.* 20 1165–1176. 10.1097/mib.0000000000000059 24874458 PMC6249688

[B83] Wurbel M. A. Malissen M. Guy-Grand D. Malissen B. Campbell J. J. (2007). Impaired accumulation of antigen-specific CD8 lymphocytes in chemokine CCL25-deficient intestinal epithelium and lamina propria. *J Immunol.* 178 7598–7606. 10.4049/jimmunol.178.12.7598 17548595 PMC2564614

[B84] Wurbel M. A. McIntire M. G. Dwyer P. Fiebiger E. (2011). CCL25/CCR9 interactions regulate large intestinal inflammation in a murine model of acute colitis. *PLoS One.* 6:e16442. 10.1371/journal.pone.0016442 21283540 PMC3026821

[B85] Wurbel M. A. Philippe J. M. Nguyen C. Victorero G. Freeman T. Wooding P. (2000). The chemokine TECK is expressed by thymic and intestinal epithelial cells and attracts double- and single-positive thymocytes expressing the TECK receptor CCR9. *Eur J. Immunol.* 30 262–271. 10.1002/1521-4141(200001)30:1<262::aid-immu262>3.0.co;2-0 10602049

[B86] Xu Z. Mei F. Liu H. Sun C. Zheng Z. (2016). C-C Motif Chemokine Receptor 9 Exacerbates Pressure Overload-Induced Cardiac Hypertrophy and Dysfunction. *J Am. Heart Assoc* 5 e003342. 10.1161/JAHA.116.003342 PMC4889199 27146447

[B87] Yokoyama W. Kohsaka H. Kaneko K. Walters M. Takayasu A. Fukuda S. (2014). Abrogation of CC chemokine receptor 9 ameliorates collagen-induced arthritis of mice. *Arthritis. Res. Ther.* 16 445. 10.1186/s13075-014-0445-9 PMC4201712 25248373

[B88] Zaballos A. Gutierrez J. Varona R. Ardavin C. Marquez G. (1999). Cutting edge: identification of the orphan chemokine receptor GPR-9-6 as CCR9, the receptor for the chemokine TECK. *J. Immunol.* 162 5671–5675. 10229797

[B89] Zabel B. A. Agace W. W. Campbell J. J. Heath H. M. Parent D. Roberts A. I. (1999). Human G protein-coupled receptor GPR-9-6/CC chemokine receptor 9 is selectively expressed on intestinal homing T lymphocytes, mucosal lymphocytes, and thymocytes and is required for thymus-expressed chemokine-mediated chemotaxis. *J Exp. Med.* 190 1241–1256. 10.1084/jem.190.9.1241 10544196 PMC2195678

[B90] Zhang J. Romero J. Chan A. Goss J. Stucka S. Cross J. (2015). Biarylsulfonamide CCR9 inhibitors for inflammatory bowel disease. *Bioorg Med Chem Lett.* 25 3661–3664. 10.1016/j.bmcl.2015.06.046 26117562

[B91] Zhang Y. Han J. Wu M. Xu L. Wang Y. Yuan W. (2019). Toll-Like Receptor 4 Promotes Th17 Lymphocyte Infiltration Via CCL25/CCR9 in Pathogenesis of Experimental Autoimmune Encephalomyelitis. *J Neuroimmune Pharmacol* 14 493–502. 10.1007/s11481-019-09854-1 31065973

[B92] Zhang Y. Liu Y. Zhu X. H. Zhang X. D. Jiang D. S. Bian Z. Y. (2014). Dickkopf-3 attenuates pressure overload-induced cardiac remodelling. *Cardiovasc Res.* 102 35–45. 10.1093/cvr/cvu004 24413772 PMC6279202

[B93] Zhong Y. Jiang L. Lin H. Li B. Lan J. Liang S. (2015). Expression of CC chemokine receptor 9 predicts poor prognosis in patients with lung adenocarcinoma. *Diagn. Pathol.* 10 101. 10.1186/s13000-015-0341-x PMC4501107 26168791

[B94] Zlotnik A. Yoshie O. (2012). The chemokine superfamily revisited. *Immunity.* 36 705–716. 10.1016/j.immuni.2012.05.008 22633458 PMC3396424

